# Cost-Utility Analysis and Value-Based Pricing of Digital Therapeutics for Pulmonary Rehabilitation in Chronic Respiratory Disease: Economic Evaluation Based on a Randomized Controlled Trial

**DOI:** 10.2196/73739

**Published:** 2025-12-15

**Authors:** Hyeonjung Park, Minseol Jang, Daehyun Kim, Chul Kim, Jun Hyeong Song, Ju Hyun Oh, Chin Kook Rhee, Jae Ha Lee, Hee-Eun Choi, Hae Sun Suh

**Affiliations:** 1 Department of Regulatory Science, Graduate School, Kyung Hee University Seoul Republic of Korea; 2 Institute of Regulatory Innovation through Science (IRIS), Kyung Hee University Seoul Republic of Korea; 3 Inje University College of Medicine Department of Rehabilitation Medicine, Inje University Sanggye Paik Hospital Seoul Republic of Korea; 4 Ajou University School of Medicine Department of Pulmonary and Critical Care Medicine, Ajou University Medical Center Suwon Republic of Korea; 5 College of Medicine, The Catholic University of Korea Division of Pulmonary and Critical Care Medicine, Department of Internal Medicine, Seoul St. Mary’s Hospital Seoul Republic of Korea; 6 Inje University College of Medicine Division of Pulmonology and Critical Care Medicine, Department of Internal Medicine, Inje University Haeundae Paik Hospital Busan Republic of Korea; 7 Inje University College of Medicine Department of Physical Medicine and Rehabilitation,Inje University Haeundae Paik Hospital Busan Republic of Korea; 8 Emocog Inc. Seoul Republic of Korea; 9 College of Pharmacy, Kyung Hee University Seoul Republic of Korea

**Keywords:** digital therapeutics, digital health therapy, DTx, economic evaluation, cost-effectiveness, cost-utility, value-based price, respiratory diseases, pulmonary rehabilitation

## Abstract

**Background:**

Pulmonary rehabilitation, a nonpharmacological treatment for chronic respiratory diseases, is underused due to limited access and time constraints. In a randomized controlled trial, the digital therapeutics (DTx) demonstrated superior efficacy to standard treatment. However, evidence on the cost-effectiveness of DTx and appropriate pricing strategies remains limited.

**Objective:**

This study aimed to evaluate the cost-effectiveness of DTx through cost-utility analysis and to explore a value-based price for its implementation.

**Methods:**

An economic evaluation was based on an 8-week randomized controlled trial involving 84 participants assigned to either the DTx group or standard treatment group. Costs were estimated from a health care system perspective. Quality-adjusted life years (QALYs) were estimated by using mapping algorithms from the chronic obstructive pulmonary disease assessment test to EQ-5D-3L. Cost-utility analysis was conducted to estimate the incremental cost-utility ratio (ICUR), which represents the additional cost per QALY gained. The willingness-to-pay threshold was set at US $19,410 per QALY. Sensitivity analyses included probabilistic sensitivity analysis, deterministic sensitivity analysis, and subgroup and scenario analyses, including a 1-year Markov model.

**Results:**

Compared with standard treatment**,** DTx increased QALY by 0.0096 at an additional cost of US $85.33, resulting in an ICUR of US $8890 per QALY gained. The maximum value-based price for an 8-week DTx program was estimated at US $192. In probabilistic sensitivity analysis, DTx had a 60.2% probability of being cost-effective at the set willingness-to-pay threshold, with 88.6% of iterations in the northeast quadrant falling below the threshold. The deterministic sensitivity analysis showed that ICURs remained below the willingness-to-pay threshold under all tested assumptions, with the maximum ICUR (US $15,644/QALY) also staying below the threshold. Subgroup analysis confirmed cost-effectiveness in both older adults (≥65 y) and non–older adults (<65 y) populations, and in both chronic obstructive pulmonary disease and interstitial lung disease groups. The 1-year Markov model estimated an ICUR of US $4398 per QALY.

**Conclusions:**

DTx for pulmonary rehabilitation demonstrated the cost-effectiveness compared with standard treatment. These findings support its potential for improving outcomes in patients with chronic respiratory disease and provide a pricing framework to facilitate its integration into health care systems.

## Introduction

Chronic respiratory diseases (CRDs), such as chronic obstructive pulmonary disease (COPD) and interstitial lung disease (ILD), are noncommunicable diseases worldwide [1]. With an increasing trend in prevalence, it is estimated that CRD constituted 4.0 million deaths in 2019 [2]. There is an increasing burden of disease on individuals and economic impact on health care systems driven by emergency department visits and hospitalizations owing to acute exacerbation [3]. In Korea, the total costs for patients with COPD, including indirect expenses, amounted to US $1.245 billion in 2015 [4].

Pulmonary rehabilitation is a nonpharmacological treatment strategy aimed at improving the physical and emotional well-being of patients with CRD [5]. It has been shown to reduce dyspnea and improve exercise capacity and quality of life (QoL) in these patients [6]. However, despite its proven benefits, pulmonary rehabilitation remains underused worldwide and is often inaccessible to patients. Lack of caregiver awareness, specialized hospitals and professionals, and insufficient financial support are major hurdles to broader use and improved treatment adherence [7-9]. Consequently, the global need for accessible and effective rehabilitation services has increased [10].

EASYBREATH (Emocog Inc) is a digital therapeutic (DTx) designed for patients with CRD, offering a comprehensive rehabilitation program. This app includes customized aerobic exercise training, breathing retraining, and an educational program prescribed based on the 6-minute walk distance (6MWD). To enhance accessibility for older adults, the app’s user interface was optimized with large buttons, clear color distinctions, and a simplified structure of menu, allowing users to navigate key features with minimal effort.

A multicenter, randomized controlled trial (RCT) evaluated the efficacy of EASYBREATH compared with a standard treatment for patients with CRD in South Korea. The trial demonstrated that EASYBREATH significantly improved clinical outcomes, including 6MWD, and patient-reported outcomes such as the modified Medical Research Council (mMRC) score, chronic obstructive pulmonary disease assessment test (CAT) score, St George’s respiratory questionnaire (SGRQ) score, and Hospital Anxiety and Depression Scale (HADS) [11]. After 8 weeks of treatment, the increase in 6MWD was significantly greater in the DTx group (mean 57.68, SD 56.25) than in the control group (mean 21.71, SD 49.70; *P*<.001), and only the DTx group exceeded the minimum clinically important difference of 54 m [12]. To contextualize the efficacy of EASYBREATH, we compared it with the Kaia app (Kaia Health Software GmbH), another DTx developed for pulmonary rehabilitation in patients with COPD. Based on the CAT scores in RCT of the Kaia app [13], we estimated an effect size of −0.605 at 6 months using Cohen *d*. In comparison, the EASYBREATH demonstrated a larger short-term effect (Cohen *d*=−1.04 at 8 wk), suggesting stronger efficacy in patient-reported outcome.

This home-based DTx program for pulmonary rehabilitation could address the key challenges associated with traditional pulmonary rehabilitation, such as accessibility and time efficiency [11]. Economic evaluations are essential to support prescription, coverage, and reimbursement decisions when integrating effective pulmonary rehabilitation using DTx into patient care and health care systems [14]. Cost-utility analysis (CUA), which evaluates the incremental cost-utility ratio (ICUR) against established willingness-to-pay (WTP) thresholds, is a widely used method in health economics. CUA uses standard health gain measures, with quality-adjusted life years (QALYs) being commonly recommended as the preferred metric for measuring benefits [15-17]. Within this framework, value-based price (VBP) aligns the rewards for innovation with the magnitude of the benefits delivered [18]. By using ICURs derived from CUA, VBP offers a transparent and replicable approach that meets the needs of patients, clinicians, and insurers while accommodating the realities of reimbursement systems [18,19].

Despite growing interest in DTx, research on the economic evaluation of DTx for rehabilitation remains limited. In this study, we aimed to evaluate the cost-effectiveness of a DTx, known as EASYBREATH, and explore a reasonable VBP that reflects its clinical and economic value for application in patient treatment.

## Methods

### Study Design and Participants

The CUA was based on an RCT conducted in 2023, which enrolled patients with CRD from 3 clinical centers in South Korea. The inclusion criteria were (1) age older than 19 years, (2) diagnosis of CRD (such as COPD, ILD, lung cancer, asthma, or bronchiectasis), (3) a need for rehabilitation due to respiratory symptoms or difficulties in daily life, and (4) the ability to use a mobile app. The exclusion criteria comprised (1) cognitive impairment, (2) previous participation in pulmonary rehabilitation, (3) unstable cardiovascular disease, (4) walking difficulties, and (5) pregnancy. A total of 84 participants met the inclusion criteria and were included in the full analysis set. Participants were randomly assigned to the DTx group (n=43) or the control group (n=41) using a block randomization method. Patients were followed up every 4 weeks for a total of 8 weeks from the initiation of treatment. Baseline characteristics were collected at the time of hospital visit before the intervention initiation. Demographic variables, such as sex, age, and clinical outcomes (such as 6MWD, mMRC, CAT, SGRQ, and HADS score), were assessed. In addition, patients were categorized by CRD subtype. Among CRD subtypes, ILD included idiopathic pulmonary fibrosis, diffuse ILD, nonspecific interstitial pneumonitis, and other fibrotic interstitial pulmonary diseases, based on clinical categorization.

### Interventions

The DTx group participated in rehabilitation sessions 3 times a week at home and visited the clinical center once every 4 weeks for examinations. Participants received an initial hands-on training session led by a research nurse to assist with app installation and usage. Each patient was equipped with a smartwatch, and the app was installed on their mobile phones.

**Figure 1:**
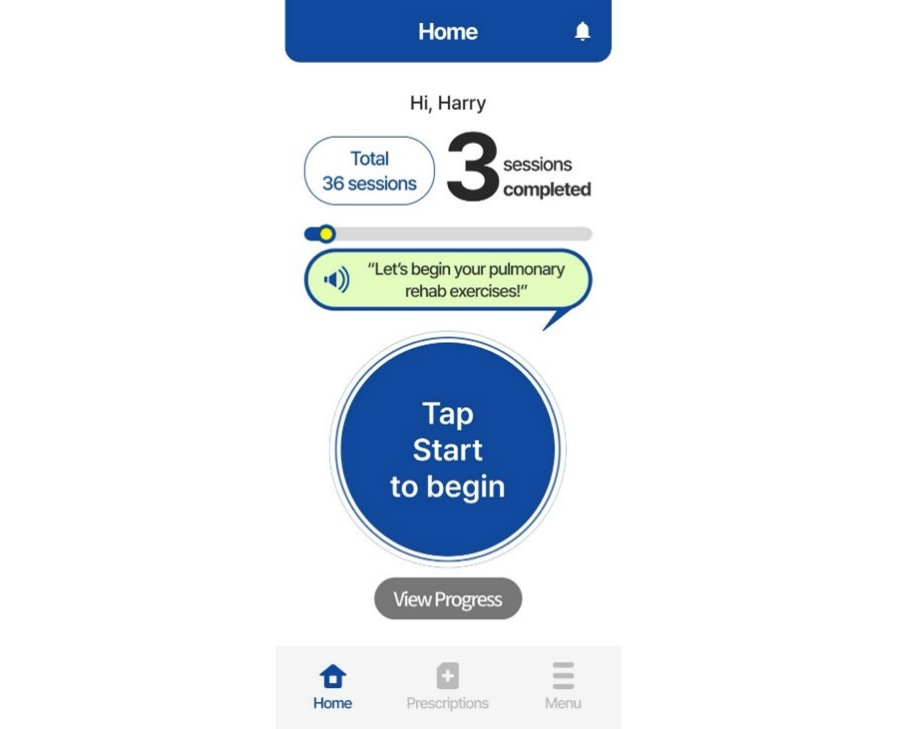
Smartphone app user interface with large fonts and a simplified design to enhance digital literacy.

To improve digital literacy, particularly among older adults, several strategies were incorporated into the trial design. These included the provision of user-friendly manuals with enlarged fonts and simplified visual instructions (Figure 1), as well as continuous monitoring by clinical staff through a web-based platform during the early stages of rehabilitation. Participants who failed to meet the minimum engagement criteria—defined as missing 3 consecutive exercise sessions—were contacted and supported by the study team. Participants’ adherence to the DTx program was monitored using a web-based platform. Adherence was calculated as the ratio of the actual number of completed sessions to the expected number of sessions during the interval between clinic visits. In addition, rehabilitation activities were documented after each session, which promoted self-awareness and supported improved adherence. Personalized exercise prescriptions were developed based on each participant’s baseline 6MWD test results, considering individual functional capacity. In addition, the DTx was integrated with a smartwatch to provide real-time feedback based on heart rate monitoring during exercise, supporting self-regulation and enhancing the safety of home-based rehabilitation.

The control group attended an educational session at the beginning of the 8-week trial and was advised to follow Korean pulmonary rehabilitation guidelines: aerobic exercise at 60% maximum intensity (3-5 times/wk) and anaerobic exercise at 60%-70% intensity (2-3 times/wk) [20]. Participants in the control group visited the clinical center every 4 weeks for scheduled assessments, identical to the DTx group.

### Cost

Costs were calculated from a health care system perspective for the base-case analysis. Two types of costs were included: (1) medical and (2) pharmaceutical costs. Expenses were collected retrospectively after the completion of the RCT and included only those directly related to CRD, as determined by clinical judgment. Medical costs, including screening, examination, and physician consultation fees, were collected based on electronic health records from the clinical trial institution. Pharmaceutical costs were calculated by summing drug acquisition costs and pharmacy dispensing fees. Drug acquisition costs were calculated using drug quantities from the electronic health record and standard unit prices from the National Health Insurance drug list [17]. For the DTx group, the cost of using the EASYBREATH app, priced at US $93.17 for the 8-week period, was included.

As the CUA did not estimate costs beyond a 12-month horizon, no discount rate was applied. All costs were initially collected in Korean won and converted to US $ using a 2023 exchange rate of US $1=1288 Korean won.

### Utility

QALY was used to assess the QoL, as it reflects both health-related QoL and life expectancy to measure overall health benefits [15]. However, the trial did not include any generic preference-based health-related QoL instruments, such as the EQ-5D, Short Form 6 Dimensions, or Health Utilities Index, which are typically required for direct utility measurement. Instead, disease-specific measures like the CAT and SGRQ were collected during the trial. These scores were mapped to utility values using validated algorithms from previous literature to estimate QALYs. In total, 3 relevant publications were identified through a literature search [21-23] (Table S1 in Multimedia Appendix 1). Among the identified algorithms, we selected algorithm by Lim et al [23] based on the following criteria: (1) lowest root mean square error and mean absolute error (Table S2 in Multimedia Appendix 1), (2) similarity in disease severity to the trial population, and (3) comparable racial characteristics (Table S3 in Multimedia Appendix 1). The study by Lim et al [23], conducted in a Korean tertiary hospital, closely aligned with the trial’s clinical context. The algorithm was internally validated using a split-sample approach and bootstrap resampling (10,000 iterations) to assess root mean square error and mean absolute error. The model with the lowest average prediction error was selected as the final algorithm [23]. Using the proposed algorithm (ordinary least squares model 3), CAT scores were mapped to EQ-5D-3L utility values. QALY was then calculated using the trapezoid rule [15].

Ordinary least squares model 3 from Lim et al [23]:

EQ- 5D- 3L utility = 1.0661 − 0.0103 × Q3 − 0.0120 × Q4 - 0.0168 × Q5 − 0.0255 × Q6 − 0.0125 × Q8

### Statistical Analysis

#### Baseline Characteristics

Baseline characteristics, including age, sex, disease type, and clinical outcomes, were collected at the time of trial enrollment. Baseline characteristics were compared between the DTx and control groups. Categorical variables were compared using the chi-square test, and continuous variables were assessed using the independent *t* test. A *P* value of <.05 was considered statistically significant.

#### Imputation of Missing Data

We collected individual patient data of full analysis set, and missing values of cost and utility measures were imputed using different methods. For missing cost data, average costs for patients at the same center were imputed. In cases where medical costs were unavailable for 1 center, average costs from the other 2 centers were used as imputed values. Missing utility values were addressed using the last observation carried forward method.

#### CUA

We conducted the CUA from a health care system perspective, including only cost items incurred within the health care system, as recommended in South Korea [6]. The result of CUA was ICUR, calculated by dividing the incremental cost by the incremental utility between 2 groups. Based on the results of the probabilistic sensitivity analysis (PSA), 95% CIs for the ICUR were estimated using the 2.5th and 97.5th percentiles of the simulation results. As no WTP threshold is specified in Korea’s health care technology assessments guidelines [17,24], thresholds are typically based on previously accepted reimbursement cases. Since the Pharmaceutical Listing System began in 2006 [7], cost-effectiveness evaluations have often referred to the 2006 per capita gross domestic product [25]. Accordingly, this study applied a WTP threshold of approximately US $19,410 per QALY to interpret the ICUR.

#### VBP

VBP is the price that reflects the comprehensive value of each intervention. We evaluated the VBP of DTx usage costs over the 8-week period. VBP was defined as the maximum price at which DTx remains cost-effective [18]. To estimate the VBP of DTx (denoted as A) compared with standard treatment (denoted as B), we used a cost-utility framework based on the ICUR. The total cost of intervention A was defined as the sum of the DTx usage cost (Fee_A_) and other health care–related costs (Othercosts_A_):

Cost_A_ = Fee_A_ + Othercosts_A_

Accordingly, the ICUR was expressed as:

ICUR = (Fee_A_ + Othercosts_A_ – Cost_B_) / (QALY_A_ – QALY_B_)

To determine the maximum VBP, the ICUR was replaced by the WTP threshold. By rearranging the equation, the VBP of DTx usage was derived as:

VBP_ Fee_A_
**=** WTP threshold × (QALY_A_ – QALY_B_) + Cost_B_ – Othercosts_A_

In addition, the usage costs were varied while holding QALYs constant to evaluate cost-effectiveness at different pricing levels.

#### Sensitivity Analysis

In total, 3 sensitivity analyses were conducted to identify uncertainties and heterogeneity. First, a PSA with 1000 simulations was performed to assess parameter uncertainty, assuming that costs followed a gamma distribution and utilities followed a normal distribution. A cost-effectiveness plane and cost-effectiveness acceptability curve (CEAC) were generated to evaluate the likelihood of cost-effectiveness. Second, a 1-way deterministic sensitivity analysis (DSA) was performed by adjusting key parameters, including costs and utilities, within 95% CIs for both groups and by re-estimating utilities using alternative mapping algorithms. Variations in ICUR were presented using a tornado diagram. Third, subgroup analysis was conducted to account for potential participant heterogeneity. Subgroups were defined by age (≥65 y and <65 y), CRD type (COPD and ILD), and analysis set (per-protocol set [PPS]), and CRD type (COPD and ILD). Data were analyzed using Microsoft Excel version 365 and SAS version 9.4 (SAS Institute Inc).

#### Scenario Analysis

To evaluate the robustness of the base-case findings, 3 scenario analyses were performed under varying assumptions. First, a limited societal perspective, as defined in the Korean guideline [17], was applied. This perspective incorporates indirect costs related to caregiving and transportation costs while excluding productivity loss costs. Informal caregiving costs were estimated using outpatient visit frequency, caregiver attendance and duration (based on opinion from a clinical expert), average hourly wages, and employment rates obtained from the Korean Statistical Information Service. Transportation costs were derived from unit transport fares reported in the Korea National Health and Nutrition Examination Survey. Second, outliers were excluded using the IQR method to examine the impact of extreme values on the cost-effectiveness results. Third, a Markov model was constructed to evaluate the long-term cost-effectiveness. This model consisted of 5 health states—“normal,” “mild,” “moderate,” “severe,” and an absorbing “death” state—defined using mMRC scores collected during the trial. The model used a 4-week cycle length and a 1-year time horizon. Transition probabilities were estimated from observed changes during the 8-week trial. State-specific costs and utilities were derived from the patient-level data using the same methods as in the base-case analysis. Detailed model specifications are provided in Multimedia Appendix 2.

### Ethical Considerations

The trial protocol was approved by the Korean Ministry of Food and Drug Safety (Approval 1431). This 8-week period RCT was approved by the Institutional Review Boards at each clinical center (Catholic University Seoul St Mary’s Hospital, KC23DNDS0031; Inje University Sanggye Paik Hospital, SGPAIK 2022-11-022-001; and Inje University Haeundae Paik Hospital, HPIRB 2022-12-013-002). Written informed consent was obtained from all participants during the primary clinical trial. For the economic evaluation study, the waiver of additional informed consent was approved, considering the retrospective study design and minimal risk to participants.

The dataset used in this study was fully deidentified and did not contain any personally identifiable information. Only patient identification codes, treatment group assignment, sex, and age were collected. No additional data that could allow for the reidentification of individual patients were included. This study did not use any images or materials containing identifiable information of individual participants. In an economic evaluation study, no additional compensation was provided.

## Results

### Participant Characteristics

A total of 84 patients were included in the randomized trial. The baseline characteristics of the patients were comparable, except for age. Table 1 illustrates the demographic characteristics of the study population. Male participants comprised 79.1% (34/43) and 87.8% (36/41) of the DTx and control groups, respectively. The mean age in years was slightly lower in the DTx group (mean 63.4, SD 10.36 y) compared with the control group (mean 67.78, SD 6.93 y; *P*<.05). COPD was the most common diagnosis, with 26 (60.47%) patients in the DTx group and 23 (56.1%) patients in the control group. Baseline clinical efficacy measures, including the 6MWD, mMRC, CAT scores, SGRQ subscales, and HADS, were well matched across the groups (all *P*>.05).

**Table 1:** Baseline characteristics of patients with chronic respiratory diseases in an 8-week randomized controlled trial evaluating a digital therapeutic for pulmonary rehabilitation.

Characteristics			DTx^a^ group (n=43)		Control group (n=41)		Total (N=84)		*P* value^b^
Male, n (%)			34 (79.07)		36 (87.8)		70 (83.3)		.44
Age, mean (SD)			63.40 (10.36)		67.78 (6.93)		65.54 (9.07)		.026
**Diagnosis, n (%)**									
	COPD^c^	26 (60.47)		23 (56.1)		49 (58.3)		.85	
	Interstitial lung disease	13 (30.23)		12 (29.27)		25 (29.8)		.92	
	Others	4 (9.3)		6 (14.63)		10 (11.9)		.70	
**Clinical efficacy, mean (SD)**									
	6MWD^d^ (m)	495.67 (64.15)		474.54 (73.36)		485.36 (69.20)		.16	
	mMRC^e^	1.30 (0.71)		1.29 (0.68)		1.30 (0.69)		.95	
	CAT^f^-total score	17.67 (6.03)		17.63 (7.39)		17.65 (6.69)		.98	
	SGRQ^g^-total	30.06 (12.90)		28.45 (13.99)		29.28 (13.38)		.58	
	SGRQ-symptoms	43.57 (13.08)		47.49 (13.91)		45.49 (13.55)		.19	
	SGRQ-activity	46.72 (21.59)		41.82 (16.89)		44.33 (19.48)		.25	
	SGRQ-impacts	16.32 (12.87)		14.86 (15.55)		15.61 (14.17)		.64	
	HADS^h^-total	9.12 (5.62)		9.98 (5.83)		9.54 (5.71)		.49	
	HADS-anxiety	3.74 (3.40)		3.78 (2.74)		3.76 (3.08)		.96	
	HADS-depression	5.37 (2.95)		6.20 (3.89)		5.77 (3.45)		.28	

^a^DTx: digital therapeutics.

^b^For continuous variables, an independent samples *t* test was used to calculate the *P* value, while for categorical variables, a chi-square test was applied. Statistical significance was assessed at a 5% significance level.

^c^COPD: chronic obstructive pulmonary disease.

^d^6MWD: 6-minute walk distance.

^e^mMRC: modified Medical Research Council dyspnea scale.

^f^CAT: chronic obstructive pulmonary disease assessment test.

^g^SGRQ: St George’s respiratory questionnaire.

^h^HADS: Hospital Anxiety and Depression Scale.

### Patient Adherence

Treatment adherence in the DTx group was assessed among participants included in the PPS at both the week 4 (n=43) and week 8 (n=40) visits. All participants demonstrated an adherence rate of ≥80% throughout the intervention period, corresponding to the “very high compliance” category [11].

#### Cost

Total costs per patient were US $390.40 (SD 193.09) for the DTx group and US $305.06 (SD 220.18) for the control group. In the DTx group, medical costs were US $196.44 (SD 37.28) and pharmaceutical costs were US $193.96 (SD 190.70). In comparison, the control group incurred medical costs at US $94.70 (SD 38.02) and pharmaceutical costs at US $210.36 (SD 227.57)

#### Utility

The mean QALY over the 8-week period was 0.1522 (SD 0.016) across all patients (Table 2). During the first 4 weeks, the QALY was 0.0822 (SD 0.010) in the DTx group and 0.0795 (SD 0.011) in the control group. In the subsequent 4 weeks, the DTx group showed significant improvement, with a QALY of 0.0746 (SD 0.016), compared with 0.0678 (SD 0.013) in the control group (*P*=.04). Over the entire 8-week period, the QALY was 0.1568 (SD 0.016) in the DTx group and 0.1473 (SD 0.013) in the control group, showing a notable difference between the groups (*P*=.005).

**Table 2:** Quality-adjusted life years across 3 time intervals (week 0-4, week 4-8, and week 0-8) in an 8-week randomized controlled trial.

Time interval	DTx^a^ group (n=43), mean (SD)	Control group (n=41), mean (SD)	Total (N=84), mean (SD)	*P* value^b^
QALY^c^ (week 0-week 4)	0.0822 (0.010)	0.0795 (0.011)	0.0809 (0.011)	.25
QALY (week 4-week 8)	0.0746 (0.016)	0.0678 (0.013)	0.0713 (0.015)	.04
QALY (week 0-week 8)	0.1568 (0.016)	0.1473 (0.013)	0.1522 (0.016)	.005

^a^DTx: digital therapeutics.

^b^An independent samples *t* test was used to calculate the *P* value. Statistical significance was assessed at a 5% significance level.

^c^QALY: quality adjusted life year.

#### CUA

Table 3 presents the results of CUA from a health care system perspective. ICUR was US $8890 per QALY (95% CI –US $29,927 to US $18,602) for 8 weeks. The DTx group incurred a higher cost of approximately US $85 but achieved an incremental QALY gain of 0.0096 compared with the control group. The ICUR was below the WTP threshold of US $19,410 per QALY.

**Table 3:** Base case cost-utility analysis of the digital therapeutics group compared with the control group during the 8-week randomized controlled trial.

Category		DTx^a^ group (n=43)	Control group (n=41)	Difference^b^
**Costs**				
	Medical costs	196.44	94.70	101.74
	Pharmaceutical costs	193.96	210.36	–16.40
	Total costs (US $)	390.40	305.06	85.34
**Utility**				
	Total QALY^c,d^	0.157	0.147	0.0096
**CUA Result^e^**				
	ICUR^f^ (US $/QALY)	8890	—^g^	—

^a^DTx: digital therapeutics.

^b^Difference was calculated by subtracting the control group value from the digital therapeutics group value.

^c^QALY: quality adjusted life year.

^d^Reflects the cumulative health utility during the 8-week trial period.

^e^CUA: cost-utility analysis.

^f^ICUR: incremental cost-utility analysis.

^g^Not applicable.

#### VBP Approach

**Figure 2:**
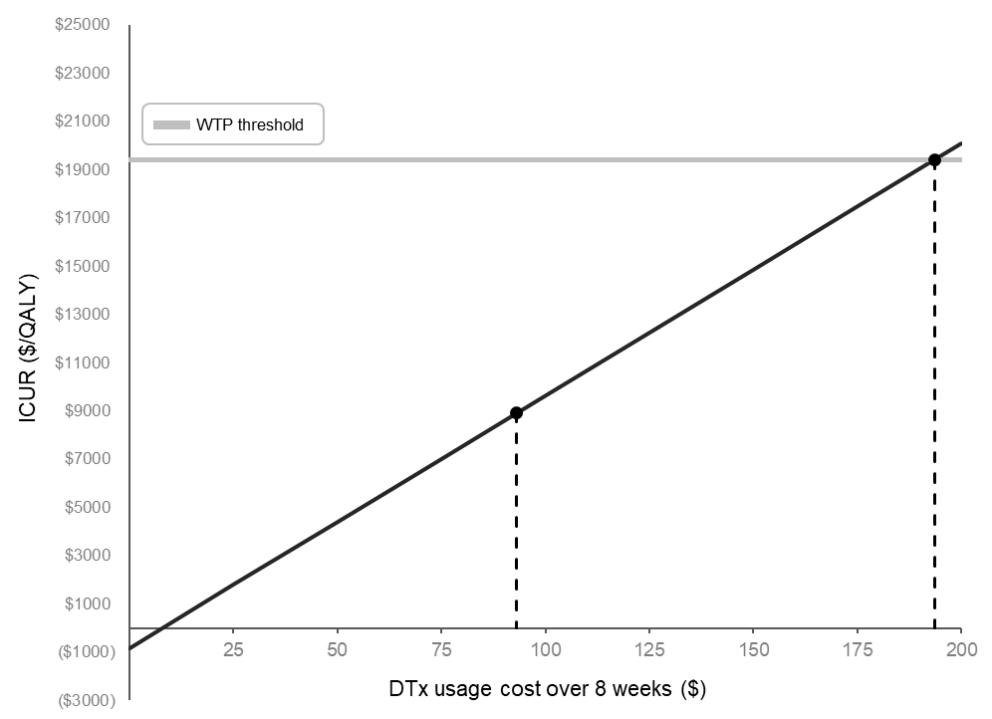
Value-based price analysis of digital therapeutics (DTx) for pulmonary rehabilitation over an 8-week period. The black line represents the incremental cost-utility ratio corresponding to the DTx usage cost. The gray horizontal line indicates the willingness-to-pay threshold (US $19,410/QALY). The black dot at US $93.17 represents the base-case DTx usage cost, whereas the intersection of the black line and willingness-to-pay threshold marks the maximum cost-effective usage cost (US $192). DTx: digital therapeutics; ICUR: incremental cost-utility analysis; QALY: quality adjusted life year; WTP: willingness-to-pay.

We estimated ICUR at different DTx usage costs for an 8-week period, which were set at up to US $200 (Figure 2). At a base-case usage cost of US $93.17, the ICUR was US $8890 per QALY, remaining below the WTP threshold (US $19,410). When the DTx usage cost increases to US $192, ICUR crossed the WTP threshold. Thus, the maximum cost-effective price for DTx usage appeared to be US $192.

#### Sensitivity Analysis

**Figure 3:**
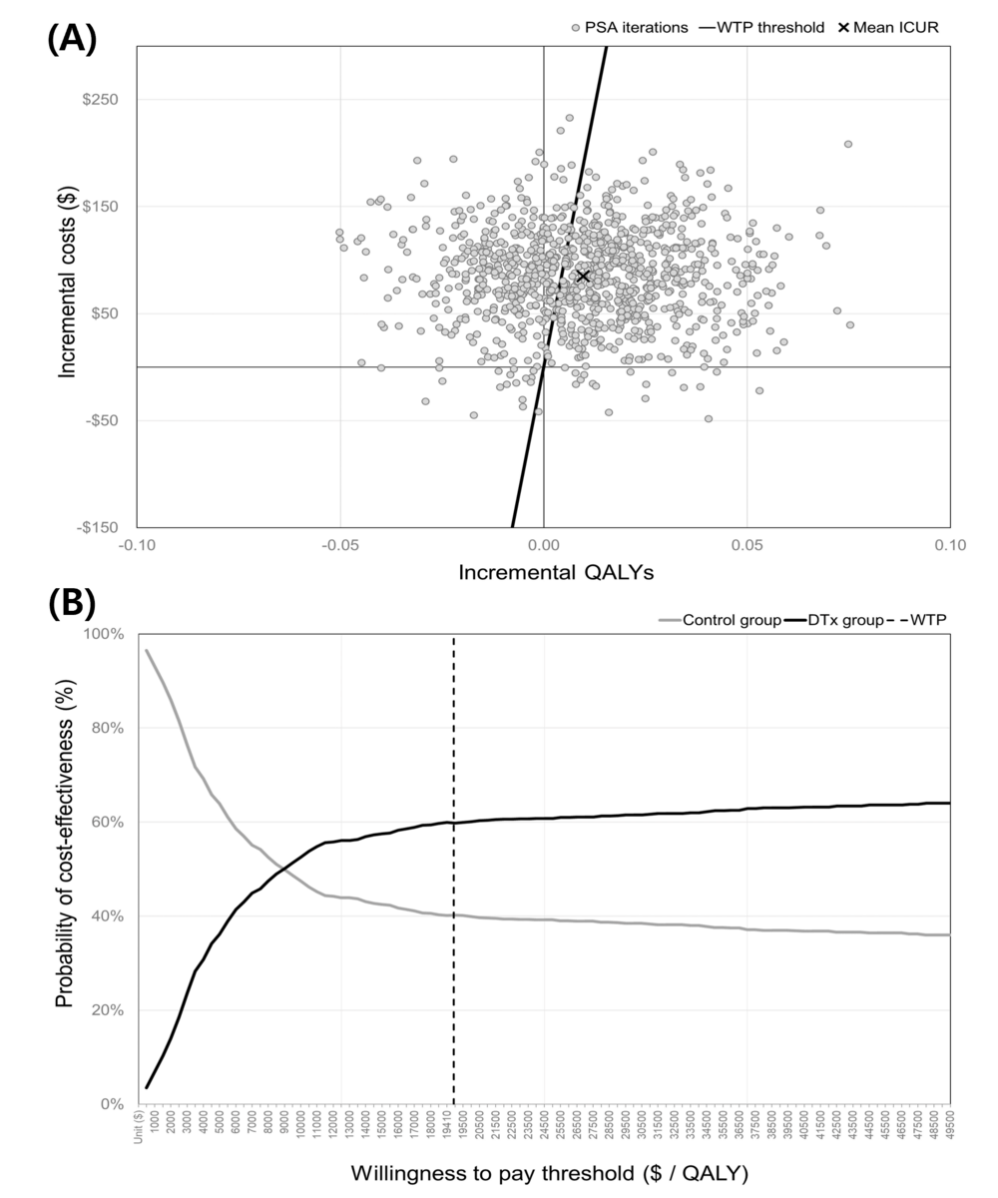
Probabilistic sensitivity analysis. (A) Cost-effectiveness plane: the scatter plot represents the distribution of incremental cost-utility ratios generated from 1000 probabilistic sensitivity analysis iterations. The black line represents the willingness-to-pay (WTP) threshold, while the “X” denotes the mean incremental cost-utility analysis. The percentages indicate the proportion of iterations in each quadrant. (B) Cost-effectiveness acceptability curve: the curve shows the probability of cost-effectiveness for the digital therapeutics group (black line) and control group (gray line) across various WTP thresholds. The dashed vertical line indicates the WTP threshold used in the base case analysis. DTx: digital therapeutics; ICUR: incremental cost-utility analysis; PSA: probabilistic sensitivity analysis; QALY: quality adjusted life year; WTP: willingness-to-pay.

In the results of 1000 simulations, the majority of ICUR (656/1000, 65.6%) were distributed in the northeastern quadrant of the cost-effectiveness plane, indicating that DTx was costlier but potentially cost-effective. Among the northeastern quadrant, 88.6% (886/1000) were below the WTP threshold. Additionally, 1.7% (17/1000) of ICUR were distributed in the southeastern quadrant, indicating that DTx was less costly and more effective (Figure 3A). The CEAC showed that DTx was more cost-effective than standard treatment (Figure 3B). At the specified WTP threshold (US $19,410 per QALY), the probability of cost-effectiveness was 60.2% in the DTx group and 39.8% in the control group.

**Figure 4:**
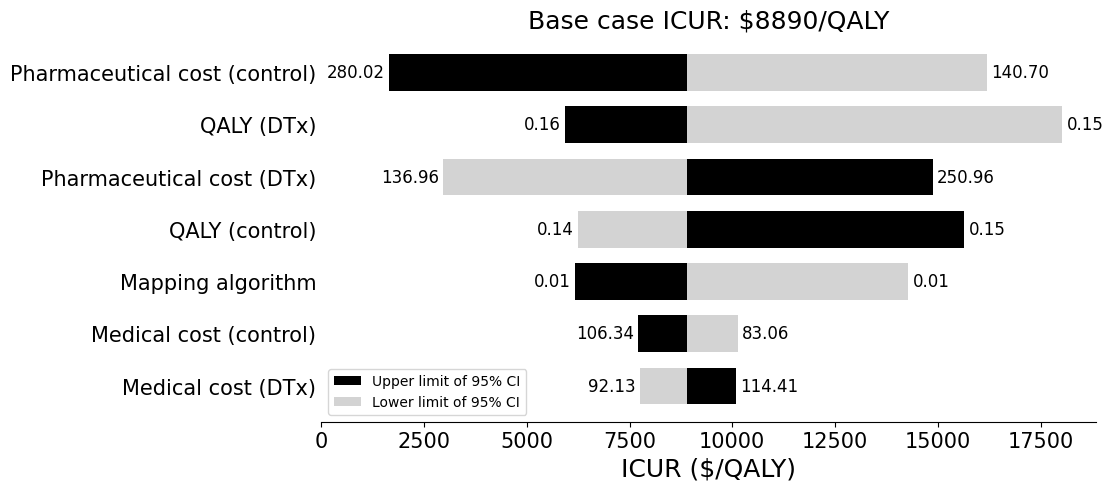
One-way deterministic sensitivity analysis (tornado diagram). Bars represent the range of incremental cost-utility ratios corresponding to the 95% CIs of each parameter. For the “Mapping algorithm” parameter, the range reflects the maximum and minimum incremental utilities derived from the alternative mapping study. DTx: digital therapeutics; ICUR: incremental cost-utility analysis; QALY: quality adjusted life year.

One-way DSA showed that the ICUR was most sensitive to the lower limit of the 95% CI for QALY in the DTx group (Figure 4). When the utility was mapped using different algorithms, the ICUR ranged from US $6178 to US $14,283 per QALY.

The results of the subgroup analysis are illustrated in Table 4. The ICUR in the PPS group was US $7275 per QALY. By age, both younger than 65 years (US $6784 per QALY) and older than 65 years age groups (US $10,486 per QALY) had ICUR values below the WTP threshold. For patients with COPD, ICUR was US $19,134 per QALY, whereas that for patients with ILD was US $559 per QALY.

**Table 4:** Subgroup analysis by study population characteristics.

Subgroup			Participants, n	Total cost (US $)	ΔCost^a^	Total QALY^b^	ΔQALY^a^	ICUR^c^ (US $/QALY)
**Base case**					85.34		0.0096	8890
	DTx^d^		43	390.40		0.157		
	Control		41	305.06		0.147		
**PPS^e^**					80.41		0.0110	7275
	DTx		36	388.17		0.159		
	Control		33	307.76		0.148		
**Age**								
	≥**65**				95.16		0.0091	10,486
		DTx	21	402.91		0.158		
		Control	29	307.75		0.148		
	**<65**				79.88		0.0118	6784
		DTx	22	378.45		0.156		
		Control	12	298.56		0.144		
**Type of CRD** ^f^								
	**COPD^g^**				122.18		0.0064	19,134
		DTx	26	336.04		0.160		
		Control	23	213.86		0.153		
	**ILD^h^**				9.15		0.0164	559
		DTx	13	526.06		0.156		
		Control	12	516.91		0.139		

^a^Difference was calculated by subtracting the control group value from the digital therapeutics group value.

^b^QALY: quality-adjusted life year.

^c^ICUR: incremental cost-utility analysis.

^d^DTx: digital therapeutic.

^e^PPS: per-protocol set.

^f^CRD: chronic respiratory disease.

^g^COPD: chronic obstructive pulmonary disease.

^h^ILD: interstitial lung disease.

In scenario analysis, the ICUR was US $9042 per QALY from a limited societal perspective. The corresponding incremental cost was US $86.80, which was only US $1.46 higher than the base-case result derived from a health care system perspective (Table 5). Based on the IQR method, outliers were identified only in pharmaceutical costs, with 7 participants (4 in the DTx group and 3 in the control group) exceeding the upper bound. After excluding these outliers, the ICUR decreased by approximately 24%, from US $8890 to US $6749 per QALY (Table 5). In the Markov model analysis, the health state distribution in the DTx group reached a steady state after cycle 7 (week 28), while continued changes were observed in the control group. Over the 1-year time horizon, the incremental cost was US $1444 and the incremental utility was 0.335, resulting in a further decrease in the ICUR to US $4580 per QALY (Table 5).

**Table 5:** Scenario analysis of cost-utility outcomes.

Scenario			n		Total cost (US $)		ΔCost^a^		Total QALY^b^		ΔQALY^a^		ICUR^c^ (US $/QALY)
**Base case**							85.34				0.0096		8890
	DTx^d^	43		390.40				0.157					
	Control	41		305.06				0.147					
Limited societal perspective							86.80				0.0096		9042
	DTx^d^	36		431.57				0.157					
	Control	33		344.76				0.147					
Excluding outliers							64.79				0.0096		6749
	DTx	32		341.42				0.159					
	Control	30		276.63				0.148					
Markov model analysis							1444				0.335		4310
	DTx	36		5116				12.012					
	Control	33		3672				11.677					

^a^Difference was calculated by subtracting the control group value from the digital therapeutics group value.

^b^QALY: quality-adjusted life year.

^c^ICUR: incremental cost-utility analysis.

^d^DTx: digital therapeutics.

## Discussion

### Principal Results

This economic evaluation demonstrated that DTx improved QALYs in patients with CRD over an 8-week period. The ICUR was US $8890 per QALY, with a 95% CI ranging from –US $29,927 to US $18,602, which falls within the cost-effectiveness threshold. In addition, the VBP analysis indicated that DTx remained cost-effective up to a usage cost of US $192 based on the estimated QALY gain. Although the CI was wide, its upper bound remained below the WTP threshold (US $19,410 per QALY). This suggests that the DTx is likely to be cost-effective under most iterations. However, the wide interval and inclusion of zero in ICUR also indicate some degree of uncertainty, highlighting the need for additional sensitivity analyses.

To explore this uncertainty, PSA and DSA were conducted. In the PSA, 30.9% of ICUR were located in the northwest quadrant, indicating that DTx was more costly and less effective on the cost-effectiveness plane. Although this suggests a risk of the DTx being dominated, 58.1% of ICUR fell below the WTP line in the northeast quadrant on the plane. Furthermore, the CEAC indicated that over half of the iterations supported the cost-effectiveness of DTx at the selected WTP threshold. The DSA indicated that the ICUR was most sensitive to variation in pharmaceutical costs in the control group, likely due to underlying differences in concomitant medications across participants. These differences might be explained by variations in the initial CRD diagnoses, as patients with different conditions required different sets of medications. The tornado diagram presented that none of the results, including those using alternative mapping algorithms, exceeded the WTP threshold.

In the clinical trial, significant differences in QALY between groups were observed after week 4. This may have resulted from both the individual effects of DTx after an initial adaptation period and the cumulative benefits of exercise. This pattern aligns with major clinical guidelines, which recommend a minimum of 6 weeks of pulmonary rehabilitation to achieve clinical benefits [23,26]. The RCT was designed to capture such delayed effects. Furthermore, such time-dependent effects should be taken into account in real-world clinical settings and in future RCTs evaluating DTx.

In this study, both groups attended follow-up visits every 4 weeks, which may have introduced a Hawthorne effect. According to clinical experts, patients with CRD generally visit hospitals every 12 weeks for routine prescriptions, suggesting that both groups were more closely monitored than would typically occur in real-world settings. However, the therapeutic effect might be maintained even in real-world practice because of continuous feedback and motivation through the app. In contrast, the standard treatment group may have been more influenced by frequent follow-up visits during the trial. Therefore, the outcomes observed in this study could be interpreted as relatively conservative estimates of the DTx’s real-world effectiveness.

With regard to cost components, the SD of pharmaceutical costs exceeded that of medical costs. Variability in pharmaceutical costs arose from differences by patient in concomitant medication and its unit price, while other medical costs are more homogeneous because of the regular schedules in the trial. Given the skewed nature of cost data in economic evaluations [27], the observed variation in pharmaceutical costs was considered acceptable. In the DTx group, although smartwatch integration was possible, it was not mandatory. Therefore, the cost of the smartwatch was not included in the DTx-related cost components.

To examine the cost-effectiveness across population heterogeneity, the subgroup analysis was conducted. DTx was cost-effective across all age groups, suggesting that the user-friendly interface and in-person training sessions effectively addressed differences in digital literacy. Additionally, DTx demonstrated particularly high cost-effectiveness among patients with ILD. The ICUR was substantially lower in the ILD group at US $559 per QALY compared with US $19,134 per QALY in the COPD group. Patients with ILD had higher baseline disease severity and incurred greater medical costs, which may have allowed more room for clinical and economic improvement. Although the cost of DTx was the same across groups, the ILD subgroup achieved larger QALY gains and greater reductions in health care costs, resulting in a more favorable ICUR. These findings suggest that disease characteristics may influence the cost-effectiveness of DTx. However, given the small sample size in the subgroup, these results should be interpreted with caution.

Additionally, when analyzed from a limited societal perspective, the incremental cost difference was minimal (US $1.46) because the unit costs for transportation and informal caregiving were identical between groups. The number of hospital visits influenced the cost difference because of its alignment with the protocol-specified schedule.

A Markov model was developed to estimate long-term outcomes. Over a 1-year time horizon, the model projected higher cumulative costs (US $1443) and QALY gains (0.3354) compared with the base-case analysis. Since the model applied a fixed transition probability observed during the trial, it implicitly assumed that the treatment effect remained constant over time. This assumption may have led to an overestimation of the incremental QALY gain and a corresponding underestimation of the ICUR (US $4302 per QALY). Therefore, these results should be interpreted with caution, given the overprojection of long-term treatment effects.

### Comparison With Previous Studies

Previous studies have shown that telemedicine-based pulmonary rehabilitation programs are more cost-effective than traditional programs [28-31]. Although telerehabilitation is a part of digital health technology, it differs from software medical device (DTx), which has clinically proven efficacy and safety [32]. There are economic evaluations of telerehabilitation [33], but no study has specifically evaluated the cost-effectiveness of DTx in pulmonary rehabilitation.

Economic evaluation studies of DTx for managing other chronic diseases such as hypertension, chronic heart failure, and type 2 diabetes have demonstrated its superior cost-effectiveness compared with standard care or telehealth interventions [34-36]. Although the characteristics of each condition and DTx differ, mobile software apps have demonstrated their potential as important tools for managing chronic diseases. These findings are consistent with our study on DTx in patients with CRD, further supporting the cost-utility of DTx in managing chronic conditions.

In South Korea, several studies have used cost-benefit analysis to evaluate telemedicine, artificial intelligence–based health care services, and mobile health care programs [37,38]. However, cost-benefit analysis is less preferred in health care technology assessments because of financial equity concerns arising from variations in WTP [39]. In contrast, CUA uses the more widely accepted QALY metric, providing acceptable evidence for health care decision-making.

South Korea operates a single-payer health care system, but reimbursement for any DTx has not yet been implemented. Nonetheless, active policy discussions are ongoing regarding DTx coverage and pricing. In this context, the US $192 VBP identified in this study for an 8-week session is considered relatively moderate, compared with in-person pulmonary rehabilitation sessions available in Korea. For comparison, the reimbursement rate for conventional pulmonary rehabilitation for CRD is approximately US $1377 for an 8-week course, which is higher than the proposed VBP for DTx. Internationally, Germany provides an example of negotiated pricing for digital health applications. For example, the Somnio app (mementor DE GmbH) was approved at 52% of the manufacturer’s proposed price (€224.92 in 90 days, as of 2022) [40]. Although DTx is not yet publicly reimbursed in Korea, the findings from this CUA and VBP analysis can serve as evidence for assessing the reimbursement feasibility of DTx.

### Implications

To the best of our knowledge, this is the first study to evaluate the cost-effectiveness of DTx for pulmonary rehabilitation and its pricing, with a focus on the value. The analysis was conducted from a health care system perspective, focusing on direct medical costs relevant to decision-makers. These results suggest that a DTx-based rehabilitation program is a cost-effective option for both patients and insurers. This study provides evidence supporting the feasibility of implementing DTx in the Korean health care environment. To support sustainable adoption within the health care system, appropriate pricing strategies are essential.

In Korea, discussions are ongoing regarding how to incorporate DTx pricing into the existing market framework. Given that the Korean health care system operates under a fee-for-service structure and a single-payer insurance system, there is limited flexibility for introducing new types of payment schemes. In this context, we adopted a VBP approach to propose a price that reflects the health outcomes gained.

However, diverse health care systems may require tailored pricing strategies in economic evaluations to reflect the economic value of DTx. For instance, subscription-based pricing models should consider both the number of eligible patients and real-world usage rates to avoid price overestimation. Outcome-based pricing requires clearly defined clinical end points and infrastructure for performance monitoring, which could be supported by integration with wearable technologies with DTx. Tiered pricing reflects differences in disease severity, adherence, or expected benefit, and it allows high-value patient groups to be prioritized through differentiated prices. Once integrated into the health care system, pricing strategies should align with both the structure of the health care system and technological characteristics of DTx—such as outcome measurability and variability in patient usage—to ensure its sustainable implementation.

### Strengths and Limitations

This study has several strengths. To the best of our knowledge, this is the first study in South Korea to evaluate the cost-effectiveness of DTx. The analysis was based on data from a confirmatory clinical trial, which enhances the internal validity of the findings. Additionally, the analysis used outcome data from 3 clinical centers covering a range of patients with CRDs, including those with COPD and ILD. High treatment adherence in the DTx group further supports the reliability of the observed effects. Furthermore, when the QALY gain of the DTx group was reduced in the DSA, the intervention remained cost-effective. The robustness of the results was confirmed by additional sensitivity analyses.

However, this study also has several limitations, including study design, data and statistical analysis, and real-world applicability. First, regarding study design and sample size, the study period was limited to 8 weeks, which did not capture long-term cost-effectiveness. Although a 1-year Markov model analysis was conducted, it was based on assumptions rather than observed long-term data. Further research with an extended follow-up period is needed. In addition, the small sample size of the trial may limit the generalizability of the results. However, the sample size was calculated with a 15% dropout rate in mind, and the robustness of the findings was supported through various sensitivity analyses.

Second, regarding data collection and analytical aspects, nonmedical expenses and productivity losses were excluded from the base case analysis due to limited data. As a result, the broader societal burdens were not fully captured, which may have led to an underestimation of the therapeutic value of DTx. However, given the mild disease severity of participants, the impact of productivity losses is likely to be minimal. To address this limitation, a scenario analysis from a limited societal perspective, including non–health care costs, was conducted based on consultations with clinical experts and previous literature.

For utility estimation, utility values were derived using a mapping algorithm rather than directly obtained from the EQ-5D-3L. As the RCT included only CRD-specific health-related QoL instruments (CAT, SGRQ, and HADS), we reviewed available mapping algorithms. Disease-specific instruments may be more responsive and sensitive to particular conditions [41]. However, condition-specific measures may have limitations in generalizability, as they could not fully reflect the impact of side effects and comorbidities on health valuation. In addition, condition labels and focusing effects, which result from narrower descriptive systems, may further limit their broader applicability [42]. Although we selected a mapping algorithm developed in a population similar to ours, it was based on cross-sectional data. This may limit to reflect longitudinal changes in individual health status. To address uncertainty in utility estimates, we applied various mapping algorithms in sensitivity analyses and selected those that best reflected disease severity, minimized prediction error, and aligned with the study design.

Additionally, there is a statistically significant difference in baseline age observed between the 2 groups, with the DTx group being younger on average. This difference may have contributed to the favorable outcomes observed in the DTx group. Although subgroup analyses stratified by an age threshold of 65 years confirmed that DTx remained cost-effective, baseline age was not adjusted as a covariate, which may have introduced residual confounding.

Third, concerning external validity and real-world applicability, the study design required both groups to attend routine follow-ups every 4 weeks, which may have inflated medical costs for the DTx group. However, in real-world settings, patients using DTx typically require routine follow-ups only every 6-12 weeks. Finally, the high adherence observed in the DTx group may have led to an overestimation of its efficacy. This adherence was likely influenced by continuous monitoring and feedback from study administrators and the lack of a double-blind design inherent to digital therapeutics. Furthermore, because adherence data for the control group were unavailable—as adherence in the DTx group was derived from the app’s built-in recording function—the relationship between treatment effects and adherence levels could not be assessed. Therefore, while the DTx group achieved superior outcomes under controlled conditions, such results may not be fully generalizable to real-world settings where adherence is typically lower. Nevertheless, adherence may be supported to some extent in real-world settings through clinician support on web-based platforms and the DTx’s self-recording feature, which encourages long-term engagement in rehabilitation.

### Conclusions

This study showed that the EASYBREATH app, a DTx-assisted pulmonary rehabilitation program, is more cost-effective than standard treatment. Cost-effectiveness was maintained up to a usage cost of US $192 per 8-week period. These findings support DTx-based pulmonary rehabilitation as an effective treatment option for managing patients with CRD and provide valuable insights for health care decision-makers. Further research is necessary to evaluate long-term effectiveness and sustainability.

## Appendix

Multimedia Appendix 1 Mapping algorithm selection.

Multimedia Appendix 2 Markov model specification.

## Abbreviations

6MWD: 6-minute walk distance
CAT: chronic obstructive pulmonary disease assessment test
CEAC: cost-effectiveness acceptability curve
COPD: chronic obstructive pulmonary disease
CRD: chronic respiratory disease
CUA: cost-utility analysis
DSA: deterministic sensitivity analysis
DTx: digital therapeutics
HADS: Hospital Anxiety and Depression Scale
ICUR: incremental cost-utility ratio
ILD: interstitial lung disease
mMRC: modified Medical Research Council
PPS: per-protocol set
PSA: probabilistic sensitivity analysis
QALY: quality-adjusted life year
QoL: quality of life
RCT: randomized controlled trial
SGRQ: St George’s respiratory questionnaire
VBP: value-based price
WTP: willingness-to-pay

## Data Availability

The dataset analyzed in this study is not publicly available because of participant confidentiality and data privacy regulations. However, they are available from the corresponding author on reasonable request.
