# Supplementary material for: Cost-Utility Analysis and Value-Based Pricing of Digital Therapeutics for Pulmonary Rehabilitation in Chronic Respiratory Disease: Economic Evaluation Based on a Randomized Controlled Trial
Source: J Med Internet Res. 2025 Dec 15;27:e73739. doi: 10.2196/73739 (PMC12750069; doi:10.2196/73739)
Supplement: Multimedia Appendix 1 [file jmir_v27i1e73739_app1.docx]

**Contents**

Table S1. Characteristics of studies identified in the mapping algorithm search

Table S2. Characteristics of patients included in the studies identified in the mapping algorithm search

Table S3. Evaluation of mapping algorithms reported in the studies

**Table S1. Characteristics of studies identified in the mapping algorithm search.**

|  | **Hoyle et al 2016** | **Starkie et al 2011** | **Lim et al 2019** |
| --- | --- | --- | --- |
| **Participants** | Patients with moderate to severe COPD (n=1,658) | Patients with moderate to severe COPD (n=3,640) | Patients with FEV1/FVC less than 70%  (n=299) |
| **Mapped from** | CAT | SGRQ | CAT |
| **Mapped to** | EQ-5D-3L utility score | EQ-5D-3L utility score | EQ-5D-3L utility score |
| **Tariff** | UK tariff | UK tariff | Korean tariff |
| **Model** | Ordinary Least Square  (Optimal model:  OLS 3) | Ordinary Least Square  (Optimal model:  OLS 2) | Ordinary Least Square  (Optimal model:  OLS 1, 3) |
| **Features included in the model** | OLS 3  Q3: chest tightness  Q5: activities  Q6: confidence  Q8: energy | OLS 2  SGRQ total score  male | OLS 1  CAT total score  age |
|  |  |  | OLS 3  Q3: chest tightness  Q4: breathlessness  Q5: activities  Q6: confidence  Q8: energy |
| Abbreviations: COPD, Chronic Obstructive Pulmonary diseases; FEV1, forced expiratory volume in 1 second; FVC, forced vital capacity; CAT, Chronic obstructive pulmonary disease assessment test; SGRQ, St. George’s Respiratory Questionnaire; EQ-5D-3L, European quality of life 5 dimensions 3 level version; UK, United Kingdom; OLS, ordinary least square; SD, standard deviation; NR, not reported. | | | |

**Table S2. Evaluation of mapping algorithms reported in the studies.**

|  | **Hoyle et al 2016** | **Starkie et al 2011** | **Lim et al 2019** | |
| --- | --- | --- | --- | --- |
| **Algorithm** | **OLS 3** | **OLS 2** | **OLS 1** | **OLS 3** |
| **EQ-5D-3L utility score** | | | | |
| Mean | 0.784 | 0.71 | NR | NR |
| Min | 0.388 | 0.13 | NR | NR |
| Max | 0.985 | 0.98 | NR | NR |
| **RMSE** | 0.163 | 0.172 | 0.111 | 0.109 |
| **MAE** | 0.119 | 0.124 | 0.082 | 0.079 |
| Abbreviations: EQ-5D-3L, European quality of life 5 dimensions 3 level version; OLS, Ordinary least square; NR, not reported; RMSE, Root mean squared error; MAE, Mean absolute error. | | | | |

**Table S3. Characteristics of patients included in the studies identified in the mapping algorithm search.**

|  | **Hoyle et al 2016** | | **Starkie et al 2011** | **Lim et al 2019** |
| --- | --- | --- | --- | --- |
| **Participants** | Patients with moderate to severe COPD (n=1,658) | | Patients with moderate to severe COPD (n=3,640) | Patients with FEV1/FVC less than 70%  (n=299) |
| **Male, n (%)** | 1141 (68.8) | | 2856 (78.4) | 258 (86.4) |
| **Race, n (%)** | NR^a^ | | NR^a^ | NR^b^ |
| **Age, mean (SD)** | 64.5 (8.5) | | 64.7 (8.4) | 69.16 (8.8) |
| **FEV1, mean (SD)** | NR^a^ | | NR^a^ | 55.85 (17.94) |
| **FEV1, n (%)** |  | |  |  |
| ≥ 80%  50-79%  30-49%  ≤ 29% | | NR | 0 (0)  1315 (36)  1828 (50)  497 (14) | 32 (10.7)  156 (52.4)  90 (30.2)  20 (6.71) |
| Abbreviations: COPD, chronic obstructive pulmonary diseases; SD, standard deviation; NR, not reported; FEV1, forced expiratory volume in 1 second; FVC, forced vital capacity.  ^a^Not reported; data was obtained from each clinical trial.  ^b^Not reported; data was obtained from tertiary hospital in Seoul, Republic of Korea, 2014. | | | | |
