# Supplementary material for: Cost-Utility Analysis and Value-Based Pricing of Digital Therapeutics for Pulmonary Rehabilitation in Chronic Respiratory Disease: Economic Evaluation Based on a Randomized Controlled Trial
Source: J Med Internet Res. 2025 Dec 15;27:e73739. doi: 10.2196/73739 (PMC12750069; doi:10.2196/73739)
Supplement: Multimedia Appendix 2 [file jmir_v27i1e73739_app2.docx]

**Cost-utility Analysis and Value-based Pricing of Digital Therapeutics for Pulmonary Rehabilitation in Chronic Respiratory Disease: Economic evaluation based on randomized controlled trial**

**Multimedia Appendix 2. Markov model specification**

**Contents**

Markov model structure

Markov model assumption

State transition matrix

Cost per health state input

Utility per health state input

# **Markov Model Structure**

A five-state Markov model was developed to evaluate the long-term cost-effectiveness. The model included four health states (“Normal”, “Mild”, “Moderate”, and “Severe”) and an absorbing state (“Death”) (Figure 1). Health states were defined based on modified Medical Research Council (mMRC) scores collected at baseline, 4 weeks, and 8 weeks during the trial. Although the mMRC scale provides a grading of dyspnea severity [1], it does not assign descriptive labels. Therefore, another labeling of each health state was assigned [2](Table 1).

Transitions were allowed between all mMRC-based health states in each cycle, while death was modeled as an absorbing state. The model adopted 4-week cycles over a 1-year time horizon (13 cycles), in accordance with the trial visit schedule [3].


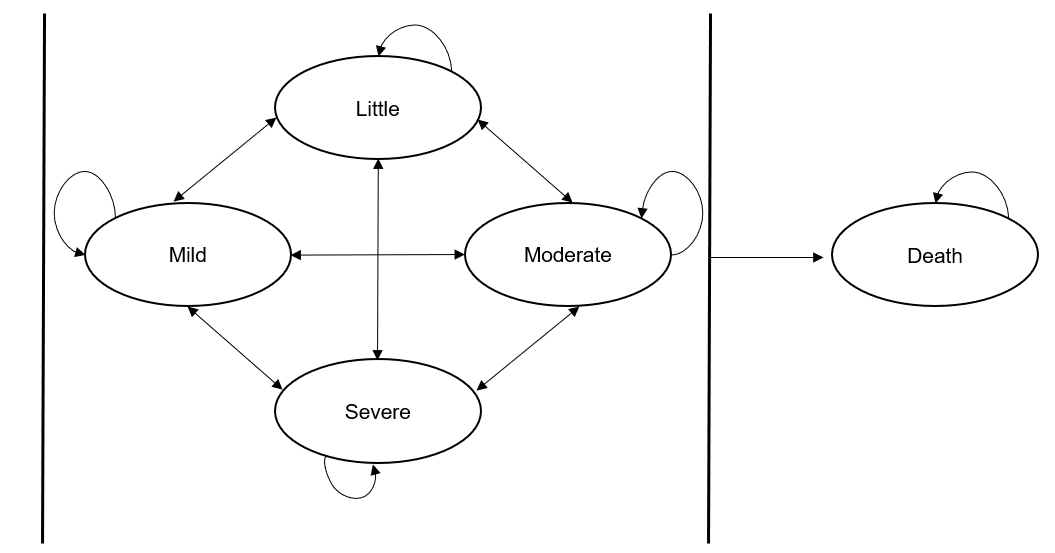


**Figure 1. Markov model state**

**Table 1. Definition of health state based on modified Medical Research Council (mMRC) score.**

| **Standard** | **Grade** | **Health state** |
| --- | --- | --- |
| Dyspnea only with strenuous exercise | 0 | Normal |
| Dyspnea when hurrying or walking up a slight hill | 1 | Mild |
| Walks slower than people of the same age because of dyspnea or has to stop for breath when walking at own pace | 2 | Moderate |
| Stops for breath after walking 100 yards (91 m) or after a few minutes | 3 | Severe |
| Too dyspneic to leave house or breathless when dressingt | 4 | Very severe |
| **Note:** The mMRC scale includes five grades (0–4); however, since no participants were classified as grade 4 in the trial, the model was constructed with four health states (excluding death). | | |

# **Model Assumptions**

- Transition probabilities (TPs) for cycles 1 and 2 (0–4 weeks and 4–8 weeks) were derived from trial data. From cycle 3 onward, the TP observed the first 4 weeks (0-4) was repeatedly applied. During trial period, this TP showed less improvement in health state transitions compared to weeks 4-8, making it a more conservative choice for long-term modeling.
- The mortality rate was based on the general Koreans at the baseline age of each group. Annual death rates obtained from Korean Statistical Information Service in 2023 were converted to cycle specific TP. A constant mortality TP was applied equally across all health states throughout the model time horizon.
- Since the model covered only a 12-month time horizon, no discount rate was applied for either costs or utilities.

# **State Transition Probabilities**

**Table 2. State transition probabilities by treatment group and cycle observed in tiral.**

| **From \ To** | **Normal** | **Mild** | **Moderate** | **Severe** | **Death** | **Total** |
| --- | --- | --- | --- | --- | --- | --- |
| **DTx group transition probability – cycle 1 (Week 0–4), cycle 3+ (Week 8 beyond)** | | | | | | |
| Normal | 0.333 | 0.333 | 0.333 | 0.000 | 0.00043 | 1.000 |
| Mild | 0.115 | 0.807 | 0.077 | 0.000 | 0.00043 | 1.000 |
| Moderate | 0.000 | 0.454 | 0.545 | 0.000 | 0.00043 | 1.000 |
| Severe | 0.000 | 0.000 | 0.666 | 0.333 | 0.00043 | 1.000 |
| Death | NA | NA | NA | NA | 1.000 | 1.000 |
| **Control group transition probability – cycle 1 (Week 0–4), cycle 3+ (Week 8 beyond)** | | | | | | |
| Normal | 0.000 | 0.999 | 0.000 | 0.000 | 0.001 | 1.000 |
| Mild | 0.000 | 0.919 | 0.080 | 0.000 | 0.001 | 1.000 |
| Moderate | 0.000 | 0.091 | 0.818 | 0.091 | 0.001 | 1.000 |
| Severe | 0.000 | 0.000 | 0.000 | 0.999 | 0.001 | 1.000 |
| Death | NA | NA | NA | NA | 1.000 |  |
| **DTx group transition probability – cycle 2 (Week 4–8)** | | | | | | |
| Normal | 0.750 | 0.000 | 0.250 | 0.000 | 0.0004 | 1.000 |
| Mild | 0.185 | 0.814 | 0.000 | 0.000 | 0.0004 | 1.000 |
| Moderate | 0.000 | 0.545 | 0.454 | 0.000 | 0.0004 | 1.000 |
| Severe | 0.000 | 0.000 | 0.000 | 1.000 | 0.0004 | 1.000 |
| Death | NA | NA | NA | NA | 1.0000 | 1.000 |
| **Control group transition probability – cycles 2 (Week 4–8)** | | | | | | |
| Normal | 0 | 0 | 0 | 0 | 0 | 0 |
| Mild | 0.037 | 0.888 | 0.074 | 0.000 | 0.0007 | 1.000 |
| Moderate | 0.000 | 0.000 | 0.999 | 0.000 | 0.0007 | 1.000 |
| Severe | 0.000 | 0.333 | 0.000 | 0.666 | 0.0007 | 1.000 |
| Death | NA | NA | NA | NA | 1.0000 | 1.000 |
| Abbreviations: DTx, Digital therapeutics; NA, not applicable. | | | | | | |

# **Health State–Specific Cost Inputs**

To estimate the cost inputs for each health state, we used patient-level cost and health state data from the trial. Based on the number of cycles each patient spent in a given state, we derived average costs per state using regression analysis. These estimates were then used as state-specific costs in the Markov model (Table 3).

**Table 3. Health state cost per cycle.**

| **Health state** | **Cost per cycle** |
| --- | --- |
| Normal | 370.19 |
| Mild | 338.70 |
| Moderate | 366.52 |
| Severe | 320.08 |
| Note: In the digital therapeutic (DTx) group, a DTx cost of $46.58 was additionally applied per cycle. | |

# **Health State–Specific Utility Inputs**

Utility for each health state was estimated by grouping patient-level EuroQol-5 Dimensions-3-Levels (EQ-5D-3L) values according to health states. EQ-5D-3L values were calculated using the same method as in the base-case analysis. These utility values were used in the Markov model to calculate quality-adjusted life years (QALYs) (Table 4).

**Table 4. Health state utility estimated from EQ-5D-3L**

| **Health state** | **Utility** |
| --- | --- |
| Normal | 0.980 |
| Mild | 0.929 |
| Moderate | 0.889 |
| Severe | 0.844 |
| Death | 0.000 |

# **References**

1. Global Initiative for Chronic Obstructive Lung Disease. Global Strategy for Prevention, Diagnosis and Management of COPD: 2025 Report. Published November 15, 2024. Accessed May 1, 2025. <https://goldcopd.org/2025-gold-report/>
2. Bestall JC, Paul EA, Garrod R, Garnham R, Jones PW, Wedzicha JA. Usefulness of the Medical Research Council (MRC) dyspnoea scale as a measure of disability in patients with chronic obstructive pulmonary disease. *Thorax*. 1999;54(7):581-586. doi:10.1136/thx.54.7.581
3. Kim C, Choi HE, Rhee CK, Song JH, Lee JH. Efficacy of Digital Therapeutics for Pulmonary Rehabilitation: A Multi-Center, Randomized Controlled Trial. *Life (Basel)*. 2024;14(4):469. Published 2024 Apr 3. doi:10.3390/life14040469
